# Update on ureteroscopy instrumentation

**DOI:** 10.4103/0970-1591.70572

**Published:** 2010

**Authors:** Renato N. Pedro, Manoj Monga

**Affiliations:** AME-SBO Lithotripsy Center Coordinator, State University of Campinas UNICAMP, Santa Barbara D’Oeste, Brazil; 1 Department of Urology, Cleveland Clinic Foundation, USA

**Keywords:** Ureteroscopy, lithotripsy, stone, instrumentation, endourology

## Abstract

The authors present an objective review of what is new in endourology instrumentation, based on up-to-date scientific data gathered from meetings, state-of-art lectures and current literature. The main scope of this review is to highlight the most recommended device options for each step of an ureteroscopy, in order to offer best care to patients.

## INTRODUCTION

Ureterorenoscopy has firmed its importance in the urology surgery arsenal. The technological advances in this field, specifically the innovations in ureteroscope design, surgical technique, and accessory instrumentation, have allowed not only for broadening of diagnostic potential of ureteral and intrarenal infirmities but also it has provided the means for treating them once diagnosed.

Associated with this technological evolution is an ever-widening array of instruments available to endourologists who need to be up-to-date on the advances of the endourological devices brought in to market, in order to offer the best care to patients.

This article is an objective review of what is new in endourology instrumentation, based on state-of-art lectures, expert meetings, and current literature.

## INSTRUMENTATION

### Guide wire

The placement of a safety wire facilitates and maintains access to the upper urinary tract. Soft-tip nitinol Glidewire (Boston Scientific Corp., Natick, MA) is the safest wire for the initial access to the ureter since it is less likely to perforate and more likely to bend when a point of obstruction is encountered.[1] In contrast, the super-stiff guide wire is the least likely to slip out inadvertently and is utilized for coaxial passage of ureteral access sheaths and large caliber stents and catheters. The Sensor wire (Boston Scientific Corp., Natick, MA) is a hybrid that contains three segments: a smooth, hydrophilic distal tip for bypassing impacted ureteral stones, a kink-resistant body (nitinol core with polytetrafluoroethylene coating), and a flexible proximal tip for back-loading of the wire through the working channel of the ureteroscope; in other word it has combined the properties of a smooth guidewire with the rigidity of the supper stiff.

### Ureteral access sheath

The use of a ureteral access sheath has been demonstrated to help facilitate ureteral re-entry, decrease operative time and cost, minimize patient morbidity, and optimize overall success with intrarenal ureteroscopic surgery.[2]

Recent clinical and in vitro trials have demonstrated that the Cook Flexor sheath (Cook Urological, Bloomington, IN) was rated superior to the Applied Access Forte XE (Applied Medical, Rancho Santa Margarita, CA) with regard to the ease of placement, instrument passage, and stone extraction.[3] The Cook Flexor sheath is also more resistant to both buckling at the ureteral orifice and kinking after removal of the inner dilator,[3] and it has one of the largest inner diameters in the most common bending positions of straight and 30 degree bends, which further facilitates stone extraction compared to Boston Scientific Navigator (Boston Scientific, Natick, MA), Applied Access Forte (Applied Medical, Rancho Santa Margarita, CA), and Bard Aquaguide (Bard, Covington, GA).[3,4]

A new balloon-based ureteral access sheath combines radial balloon dilation and access sheath placement in a single step that reduces both the axial force and urothelial disruption, improving saline flow, causing less trauma to the urothelium, and showing an easy insertion in a porcine model.[5]

### Flexible ureteroscope

The newer, actively deflecting flexible ureteroscopes offer increased lower pole access compared to the older passively deflecting scopes by one of two mechanisms: either separate dual-lever primary and secondary deflection that offers increased unidirectional downward deflections of 270 degrees (Gyrus-ACMI Dur8-E, Stryker Flexvision) or increased bidirectional primary deflection that offers 270 degree deflection in both directions (Gyrus-ACMI Dur-D, Olympus URF-P5, Karl Storz Flex-X2, Richard Wolf Viper).

A comparison of commercially available flexible ureteroscopes concluded that the larger working channel of the Wolf ureteroscopes provides superior irrigant flow as well as better optics through the unique fused quartz bundle compared to glass fiberoptic bundles.[6] The Wolf Viper (Richard Wolf Endoscopy, Vernon Hills, Illinois) 7.5Fr was shown to have twofold greater resolution than the other flexible ureteroscopes, as defined by the imaging system’s ability to distinguish object detail. In addition, in vitro evaluations of scope manipulation have demonstrated that the Wolf Viper is superior at accessing all calyces in a hydronephrotic kidney model.[7]

Another critical consideration is the durability of the flexible ureteroscope. The first flexible scope durability study showed that Gyrus-ACMI DUR-8-Elite was the most durable flexible ureteroscope[8] ; however new studies on the next generation of flexible ureteroscopes demonstrated that the Wolf Viper, Olympus URF-P5, and Stryker Flexvision U-500 flexible ureteroscopes seem comparable with regard to durability, lasting longer (up to fivefold) than the previous generation.[9]

Modern digital flexible ureteroscopes (Olympus URF- Vo, ACMI DUR-D) improved maneuverability and visibility compared to the conventional fiberoptic scopes as they have eliminated the light cord and improved optical resolution with CMOS technology (complementary metal oxide semiconductor). This new generation of scopes also employs the latest in light technology the LED (light emitting diode), a durable and cheap cool light, eradicating the traditional Xenon light, a very expensive, hot, and less-durable light source. In addition, the absence of optic fibers in the shaft of the flexible scope provides better deflection and simplicity to the instrument, which lowers costs and results in more durability.[10]

### Intracorporeal lithotrite

While a number of different endoscopic lithotrites, such as ultrasonic, electrohydraulic, pneumatic, and laser have been utilized in the past, holmium laser has come to dominate intracorporeal lithotripsy. Holmium:YAG laser lithotripsy causes chemical decomposition of calculi predominately as a consequence of a photothermal mechanism to create a vaporization bubble that subsequently destabilizes the stone.[11]

The laser fibers are thin and flexible, making them ideal for passing through the working channel of a flexible ureteroscope. In contrast to other lasers, holmium laser lithotripsy has been shown to fragment all compositions of urinary calculi, as well as produce smaller stone fragments than pneumatic or electrohydraulic lithotripsy. In addition, the holmium laser energy is efficiently absorbed in a fluid medium, minimizing the risk of urothelial injury compared to the electrohydraulic lithotrite. Furthermore, retropulsion of the stone is less likely than with a pneumatic lithotrite.

In a comparison study, the stone-free rates both at the end of the ureteroscopy and 3-month post-procedure were significantly higher for holmium versus electrohydraulic lithotripsy.[12]

Performance and safety studies of commercially available holmium laser fibers demonstrated that the Dornier Lightguide 200 was the most likely of small fibers (200-273 mm) to fracture and damage a flexible ureteroscope, while the Lumenis 272 (Coherent) and the Innova Quartz 400 (Gyrus-ACMI) were the most durable in their size class.[13]

### Stone retrieval devices

A variety of stone retrieval devices are utilized in ureteroscopy under different circumstances. Important properties of these devices include visibility during stone manipulation, sufficient radial force to open in the ureter, and the ability to capture, retain, or, if necessary, disengage a stone. Alligator or rat tooth forceps are favored by some due to the reversible grasp, reusability, and corresponding cost-effectiveness. However, the large size (3F or greater) and weak grasp impact their effectiveness.[14]

Nitinol-basket based designs, however, are more versatile and atraumatic in stone retrieval due to the unique pliability of the wires and the flexibility to allow full lower pole deflection of a flexible ureteroscope in the majority of cases.[15,16] In general, in vitro studies have shown that the basket configuration and linear opening dynamics of the Cook NCircle 2.2F (Cook Urological) best facilitate efficient stone capture from ureteral and calyceal models compared to 12 other baskets.[17–19] The Cook N-Compass (Cook Urological) has a webbed configuration that best facilitates the capture of stones as small as 1 mm in size, and is used when multiple small stone fragments are present.

The 1.5F Sacred Heart Halo (Sacred Heart Medical, Minn) has been demonstrated in a calcyeal model to be the most efficient at stone retrieval of smaller fragments.[20] In addition, it allows rotation of an engaged stone via a rotary wheel on the basket handle, a technique that is utilized if a stone is too large for removal down the ureter. Furthermore, a 200-μm laser fiber can be passed alongside the Halo basket, and simultaneous laser lithotripsy/stone rotation can be performed for more complete stone fragmentation. The 1.9F Escape nitinol stone retrieval basket (Boston Scientific, Natick, MA) relies on the same concept of “side by side” approach, as it is designed to capture calculi and facilitate simultaneous laser lithotripsy *in situ*, preventing retrograde ureteral stone migration.[21] These two devices have particular interest in cases of entrapped ureteral or renal stones.

The Cook NGage is a relatively new device that has coupled the properties of a three-prong grasper with the entrapment capability of a regular tipples nitinol basket. NGage provides easy grasp-and-release of the stone, making it possible to relocate the calculus from the lower pole to the upper pole or to a straight forward path to expedite and optimize laser lithotripsy.[22]

### Ureteral occluding devices

A variety of devices are utilized to prevent stone migration during intracorporeal lithotripsy in the ureter. The Stone Cone (Boston Scientific) consists of concentric coils which, when placed proximal to calculi, act to prevent proximal retropulsion of stone fragments during lithotripsy.[23] The device has been shown clinically to reduce the incidence of residual stone fragments of over 3 mm in size. The Cook N-Trap is a 2.8F deployable “backstop” composed of 24 interwoven nitinol wires that has been shown in ex vivo pig ureters to prevent the migration of small plastic beads as small as 1.5 mm.[24]

### Ureteral stent

Ureteral stents are used for both the prevention and treatment of ureteral obstruction following ureteroscopy. A ureteral stent is always left after placement of a ureteral access sheath, as anecdotal experience with not stenting in this situation is a higher prevalence of significant transient pain for 24 hours. The Bard Inlay 6F ureteral stent (Bard Medical) has been associated with less severe urinary symptoms than other ureteral stents.[25]

Interestingly, in a recent multicenter clinical trial the use of a drug-eluting stent (anti-inflammatory - ketoralac) to decrease stent-related discomfort with promising results has been demonstrated, especially in a selected group of patients such as young adults.[26]

## EXPERIMENTAL STUDIES-FUTURE?

New endourology devices are always being idealized and some have already been tested in “in vivo” animal studies. The first noteworthy innovation was presented at the American Urological Association Meeting in Chicago and it was about the use of iron-oxide microparticles that bind to the calcium component of the stones turning them attractable to a new magnet-tip basket, expediting and optimizing stone extraction during endoscopic surgery.[27]

Another interesting study was presented at the European Association of Urology at Stockholm evaluating the use of isoproteronol (a ß 1,3 adrenoreceptors stimulant) as an intrarenal lowering pressure agent during ureteroscopy. The concept of using a pharmacological agent in the irrigating solution during ureteroscopy is of fascinating potential.[28]

These are a few of interesting ideas that can potentially result in better outcomes for the patients and facilitate the procedure for the endourologists.

## CONCLUSION

The growing prevalence of flexible ureteropyeloscopy as a diagnostic and therapeutic tool for endourologists is due in large part to the dramatic evolution in instrument design and technology. Having the right instrument in the right situation will help facilitate consistent and positive operative outcomes.
